# *AC016745.3* Regulates the Transcription of AR Target Genes by Antagonizing NONO

**DOI:** 10.3390/life11111208

**Published:** 2021-11-09

**Authors:** Yali Lu, Xuechao Wan, Wenhua Huang, Lu Zhang, Jun Luo, Dujian Li, Yan Huang, Yao Li, Yaoting Xu

**Affiliations:** 1 State Key Laboratory of Genetic Engineering, School of Life Science, Fudan University, Shanghai 200433, China; 19110700066@fudan.edu.cn (Y.L.); wanxuechaosu@126.com (X.W.); hwh_2020@163.com (W.H.); zhanglu407@fudan.edu.cn (L.Z.); huangyan@fudan.edu.cn (Y.H.); 2 Department of Urology, Shanghai Fourth People’s Hospital Affiliated to Tongji University School of Medicine, Shanghai 200434, China; abell_luojun@sina.com (J.L.); djl00777@163.com (D.L.)

**Keywords:** prostate cancer, androgen receptor, *AC016745.3*, NONO, transcriptional regulation

## Abstract

The androgen receptor (AR) and its related signaling pathways play an important role in the development of prostate cancer (PCa). Long non-coding RNAs (lncRNAs) are involved in the regulation of tumorigenesis and development, but their specific mechanism of action remains unclear. This study examines the function and mechanisms of action of lncRNA *AC016745.3* in the development of PCa. It shows that dihydrotestosterone (DHT) results in the AR-dependent suppression of *AC016745.3* expression in the LNCaP androgen-sensitive human prostate adenocarcinoma cell line. In addition, overexpression of *AC016745.3* inhibits the proliferation and migration of PCa cells, and suppresses the expression of AR target genes. This research also demonstrates that the protein NONO interacts with AR and functions as an AR co-activator, promoting AR transcriptional activity. Furthermore, using RNA immunoprecipitation (RIP)-PCR experiments, the study demonstrates that both NONO and AR can bind *AC016745.3*. Moreover, cell phenotypic experiments reveal that NONO can promote cellular proliferation and migration, and that *AC016745.3* can partially antagonize the pro-oncogenic functions of NONO in PCa cells. In summary, the results indicate that *AC016745.3* can bind NONO, suppressing its ability to promote AR-dependent transcriptional activity. Furthermore, DHT-dependent suppression of *AC016745.3* expression can enhance NONO’s promotion effect on AR.

## 1. Introduction

Prostate cancer (PCa) is a common malignancy of the male reproductive system. The incidence of prostate cancer ranks second among male malignancies worldwide [1]. The androgen receptor (AR) and AR-related signaling pathways play important roles in the development of prostate cancer. Under conditions of androgen stimulation, AR enters the nucleus and binds specific androgen response elements (AREs) within target genes, resulting in the recruitment of cofactors and the formation of protein complexes that regulate gene expression [2,3]. Androgen deprivation remains the main method for treating PCa. However, the androgen dependence of PCa cells gradually weakens over the course of androgen deprivation therapy (typically 18 to 24 months), and these cells eventually develop androgen-independence [4,5]. During androgen deprivation therapy, PCa continues to produce PSA in large quantities [6,7], indicating that the AR signaling pathway remains intact as PCa cells transition to androgen independence [8]. Under such conditions, AR activity may be sustained via ligand-independent mechanisms. The role of AR and its downstream regulatory genes in the transition of the tumor to androgen-independence remains an area of intense interest in the field of PCa research.

Long non-coding RNAs (lncRNAs) are defined as transcripts of more than 200 nucleotides that are not translated into proteins [9]. There is a growing body of evidence indicating that many lncRNAs are differentially expressed in various tumors, and that they may have a regulatory role in the emergence and development of cancer. The AR signaling pathway plays a vital role in the development of PCa. Certain lncRNAs that are differentially expressed in PCa have been shown to interact with AR [10,11]. Furthermore, recent evidence suggests that some lncRNAs, such as *PlncRNA-1*, *HOTAIR*, and *PRNCR1*, can both interact with and affect the transcriptional activity of AR [12,13,14,15].

The authors of this paper recently identified *AC016745.3* as an androgen-responsive lncRNA of unknown function that is differentially expressed in PCa. *AC016745.3* is 508 bp in length and is located within an intergenic region. This current study shows that *AC016745.3* can function as a tumor-suppressor in PCa, and that increased *AC016745.3* expression inhibits AR-dependent gene transcription in LNCaP cells. The results indicate that *AC016745.3* interacts with the AR co-activator NONO, suppressing its transcription-promoting activity towards AR. Conversely, decreased expression of *AC016745.3* in response to dihydrotestosterone (DHT) stimulation can enhance NONO’s promotion effect on AR.

## 2. Materials and Methods

### 2.1. Cell Culture, Androgen Treatment

LNCaP cells were purchased from the American Type Culture Collection (Manassas, VA, USA) which were confirmed by short tandem repeat analysis. DU145 and PC-3 were obtained from Cell Bank of Chinese Academy of Sciences (Shanghai, China) and were authenticated by mycoplasma detection, DNA-fingerprinting, isozyme detection, and cell vitality detection. LNCaP, PC-3, 22Rv1 and DU145 were cultured routinely using RPMI 1640 medium supplemented with 10% fetal bovine serum (FBS), 1% non-essential amino acids (NEAA), 1% sodium pyruvate (PS), and 1% ampicillin/streptomycin sulfate (S/P). All cells were cultured at 37 °C in 5% CO_2_.

Dehormone starvation treatment was undertaken using RPMI 1640 phenol red-free medium with 5% activated carbon adsorption-treated fetal calf serum (CCS-FBS), 1% non-essential amino acids (NEAA), 1% sodium pyruvate (PS), and 1% ampicillin/Streptomycin sulfate antibody (S/P). LNCaP cells treated with androgen after three days of hormone starvation treatment. According to the experimental requirements, the DHT treatment dose was 0.1 nM-1000 nM final concentration, the treatment time was 2–48 h, and the corresponding volume of ethanol was used as a control.

### 2.2. RNA Interference and Transfection

All small interfering RNA (siRNA) oligonucleotides against NONO and negative control (NC) were purchased from GenePharma (Shanghai, China), and used at 50 nM concentration. See Supplementary Table S3 for siRNA sequence. Transfection was carried out with Hilymax Transfection Reagent (Dojindo Laboratories, Japan) according to the manufacturer’s procedure.

### 2.3. qRT-PCR Analysis

RNA was purified with TRIzol (Invitrogen) and reverse transcribed with PrimeScript RT Master Mix (Takara) using 500 ng of RNA and 0.25 µM of random hexamers. qPCR was performed using the SYBR Green PCR Master Mix (Vazyme). The data were analyzed using the 2^−ΔΔCT^ method. Refer to Supplementary Table S4 for qPCR primers.

### 2.4. RNA Pull down Assay

The T7 promoter-containing DNA fragment was used as a template for in vitro transcription according to the product instructions (Promega, 0000359591) and followed by RNA extraction (Table S6). For labeled RNA, 2.5 nM biotin-16-dUTP (Roche) was used. An amount of 2 × 10^7^ cells were lysed with the prepared NP40 (50 × PI, 100 × PMSF, fresh 100 U RNase inhibitor/mL) to extract the nuclear proteins. The nuclear proteins were mixed with a concentration of 2 mg/mL and 50 pmol Biotin-Labeled RNA in buffer NT-2 (0.05% NP40, 50 mM Tris-HCl, 150 mM NaCl, 1 mM MgCl2) and incubated for 2 h, then 60 μL of Streptavidin agarose (Thermo Scientific, 20357) was added, and incubated for 1 h. After the beads are eluted with buffer NT-2, the target protein was identified by mass spectrometry or Western blotting analysis.

### 2.5. Mass-Spectrometric Sequencing Analysis

An Orbitrap mass spectrometer (Thermo Fisher) was used to perform mass-spectrometric sequencing with RNA pull-down protein. The sample was loaded onto a CAPTRAP column (0.5 × 2 mm, MICHROM Bioresources) in 5 min at a flow rate of 10 μL/min. The sample was subsequently separated by a C18 reverse-phase column (0.075 × 150 mm, packed with 3 μm Aeris C18 particles, Phenomenex) at a flow rate of 300 nl/min. Protein searches were performed with Mascot 2.3.02 software (MatrixScience) against the SWISSPROT human protein database (release 2014_07). The mass spectrometry proteomics data have been deposited to the ProteomeXchange Consortium via the PRIDE [16] partner repository, with the dataset identifier PXD029141.

### 2.6. RNA Immunoprecipitation Assay

An amount of 2 × 10^7^ cells were lysed with 0.5% NP40 and the supernatant was collected as a lysate by centrifugation. An amount of 50 μL of Protein-A magnetic beads was equilibrated with 5% BSA-NT2 buffer and 3–5 μg of antibody was added, then incubated overnight at 4 °C. The beads were washed 4–5 times with pre-chilled NT2 buffer, and cell lysate added and mixed well. An amount of 100 μL was taken as Input, and the remaining samples were incubated at 4 °C for 4 h. The magnetic beads were washed 4 times with pre-chilled NT2 buffer. An amount of 500 μL Trizol was added to extract RNA for qRT-PCR analysis. The following antibodies were used: anti-AR (Abcam, ab108341), anti-NONO (Proteintech, 11058-1-AP) and anti-IgG (Abcam, ab2410).

### 2.7. Chromatin Immunoprecipitation Assay

Chromatin immunoprecipitation (ChIP) was performed as described previously [17]. Chromatin immune precipitates for proteins were amplified by qPCR and then normalized to Input and calculated as percentages of Inputs. Fold enrichment levels indicated the fold changes over the NC immunoglobulin G. In this experiment, KLK3, KLK2, FKBP5, TMPRSS2, NEDD4L, CTBP1, PSA enhancer and miR-125b, which are all androgen-responsive elements (ARE), worked as the positive control, whereas XBP-1 promoter worked as the negative control. The antibodies anti-AR (Abcam, ab108341) and anti-IgG (Abcam, ab2410) were used in ChIP. Primers for ChIP-PCR are listed in Supplementary Table S5.

### 2.8. Co-Immunoprecipitation

An amount of 1 × 10^7^ cells were lysed with 0.1% NP40 and the supernatant was collected by centrifugation. The AR antibody added to the cell lysate, and incubated overnight at 4 °C on a rotating wheel. Protein A agarose beads (Millipore, 16-157) or protein G agarose beads (Millipore, 16-201) were added to the lysate and spun at 4 °C for 4 h. The centrifuge was set at 2000 rpm for 3 min, the supernatant was discarded to collect the precipitate, and washed 4 times with Wash Buffer. The supernatant buffer was added and then boiled for 10 min, and centrifuged to collect the supernatant for mass spectrometry or Western blotting analysis.

### 2.9. Cell Migration Assay

An amount of 10,000 digested cells were seeded in the upper layer of each transwell chamber of a 24-well plate, and cultured for 48–72 h. Cells were fixed with 100% methanol, and migrating cells were stained with DAPI staining solution. The number of cells that migrated to the lower layer of the chamber were observed with an Olympus inverted fluorescence microscope.

### 2.10. Cell Proliferation Assay

A cell proliferation assay was performed as described previously using Cell Counting Kit-8 (Dojindo Laboratories, Japan) according to the manufacturer’s instructions. Absorbance was measured at 450 nm with Microplate Reader ELx808 (Biotek, Winooski, VT, USA) at different time points, the absorbance at 630 nm was used as a reference.

### 2.11. Cell Cycle Assay

Cells were collected and treated with 0.03% Triton X-100 and 50 ng/mL propidium iodide (PI) for 15 min in the dark. The percentage of cells in different cell cycle phases (G1, S and G2) were measured by FACScalibur flow cytometer (BD, Lake Franklin, NJ, USA).

### 2.12. Annexin V-FITC Apoptosis Detection

Cell apoptosis was determined with the FITC-Annexin V Apoptosis Detection Kit (BD, Franklin Lakes, NJ, USA) by FACSCalibur flow cytometer (BD) according to the manufacturer’s instructions.

### 2.13. Statistical Analysis

The numerical data were presented as mean ± standard deviation (SD) of at least three determinations. Statistical comparisons between groups of normalized data were performed using T-test or Mann–Whitney U test according to the test condition. *p* < 0.05 was considered statistically significant with a 95% confidence level.

## 3. Results

### 3.1. AC016745.3 Is a Target of AR-Dependent Gene Repression and Is Downregulated in LNCaP Cells in Response to DHT Stimulation

**Figure 1:**
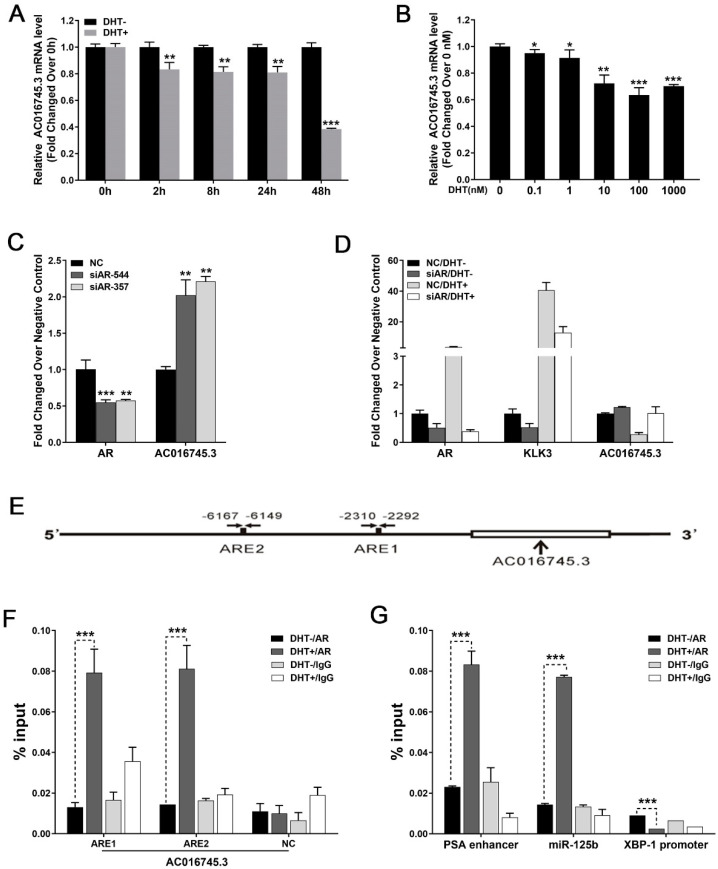
*AC016745.3* is the direct target gene of AR. (**A**,**B**) Treatment of LNCaP cells with 10 nM DHT according to time (0 h, 2 h, 8 h, 12 h, 48 h) and concentration gradient (0 nM, 0.1 nM, 1 nM, 10 nM, 100 nM, 1000 nM) treated 48 h, and qRT-PCR was used to detect the expression of *AC016745.3*. (**C**) After using *si-AR-357* and *si-AR-544* knocking down the expression level of AR in LNCaP cells, the expression changes of *AC016745.3* were detected by qRT-PCR. (**D**) Androgen deprivation treatment in LNCaP cells, the expression level of *AC016745.3* was detected after knocking down the expression level of AR. (**E**) Using Genomatix software to predict the possible ARE binding site in the 10 kb region upstream of the transcription start site (TSS) of *AC016745.3*. (**F**,**G**) ChIP-PCR experiment was used to detect the combination of AR and the predicted ARE elements of *AC016745.3*. The PSA enhancer (*KLK3*) and *miR-125b* served as a positive control, while the *XBP-1* promoter served as a negative control. The result is the percentage of DNA in the Input. Data are presented as the mean ± SD (n = 3). Significance was defined as *p* < 0.05 (*, *p* < 0.05; **, *p* < 0.01; ***, *p* < 0.001).

To identify lncRNA *AC016745.3* as an androgen-responsive RNA, LNCaP cells were treated with the androgen DHT and changes in *AC016745.3* expression were examined. These experiments revealed that DHT treatment resulted in both a dose-dependent and time-dependent decrease in the expression of *AC016745.3* (Figure 1A,B). Moreover, AR knockdown in LNCaP cells resulted in a significant upregulation in the expression of *AC016745.3*, whereas this had no such effect if the cells had previously been exposed to androgen deprivation treatment (Figure 1C,D). Together these findings demonstrate that *AC016745.3* expression is negatively regulated by AR.

To determine whether the *AC016745.3* gene is either a direct or secondary target of AR, a search for potential androgen response elements (ARE) within the *AC016745.3* gene was undertaken using Genomatix software, and two putative sites were identified (Figure 1E; ARE1: −2292/−2310, ARE2: −6149/−6167). ChIP-PCR results revealed that the predicted *AC016745.3* ARE sites were significantly enriched in AR immunoprecipitates, following the stimulation of LNCaP cells with 100 nM DHT (Figure 1F,G). These results indicate that the *AC016745.3* gene is a direct target of AR repression and that the androgen-dependent recruitment of AR to ARE sites within this gene results in the inhibition of lncRNA *AC016745.3* expression.

### 3.2. AC016745.3 Acts as a Tumor Suppressor Gene

**Figure 2:**
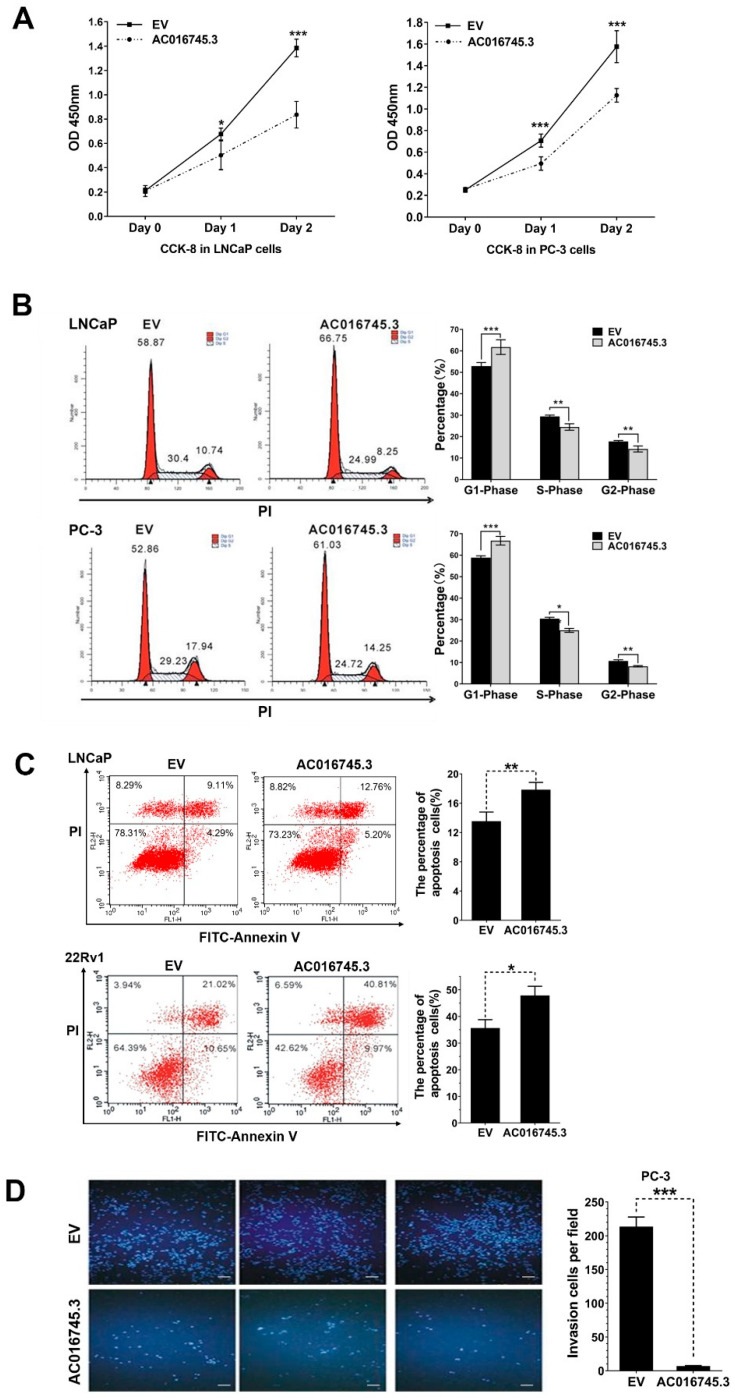
*AC016745.3* plays a tumor suppressor role in prostate cancer. (**A**) After highly expressing *AC016745.3* in prostate cancer cell lines LNCaP and PC-3, the cell growth rate changes were detected using CCK-8 kit. (**B**) After highly expressing *AC016745.3* in prostate cancer cell lines LNCaP and PC-3, stained with PI and detected the cell cycle changes by FACScalibur flow cytometry. (**C**) After highly expressing *AC016745.3* in prostate cancer cell lines LNCap and 22Rv1, stained with Annexin V/PI, and detected the changes of early and late apoptosis by FACScalibur flow cytometry. (**D**) Cell migration assay was performed in PC-3 cells by counting the amount of cells migrating in the transwell chamber after high expression of *AC016745.3*. Scale Bar = 100 μm. Data are presented as the mean ± SD (n = 3). Significance was defined as *p* < 0.05 (*, *p* < 0.05; **, *p* < 0.01; ***, *p* < 0.001).

Next, the role of *AC016745.3* in the development of PCa was explored. Cell phenotype experiments showed that *AC016745.3* overexpression inhibited the proliferation (Figure 2A), blocked the G1-to-S phase transition (Figure 2B), promoted the apoptosis (Figure 2C), and inhibited the migration (Figure 2D) of PCa cells. Taken together, these findings suggest that lncRNA *AC016745.3* plays a tumor suppressive role in PCa.

### 3.3. AC016745.3 Binds the AR Transcription Complex and Inhibits AR-Dependent Gene Transcription

**Figure 3:**
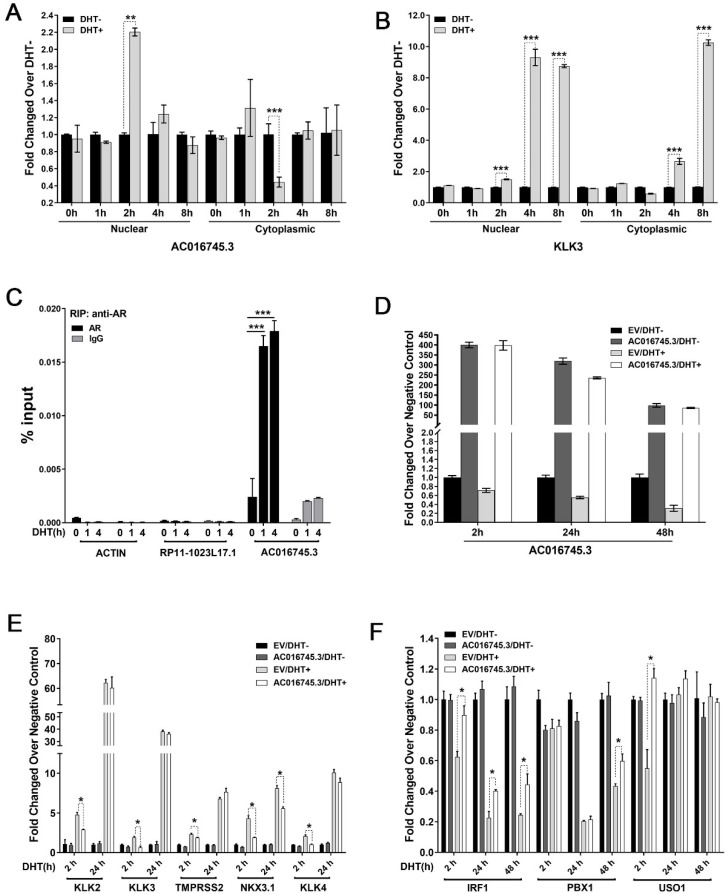
*AC016745.3* may affect the transcriptional regulation of AR on its target genes. (**A**,**B**) qRT-PCR analysis of *AC016745.3* expression in nucleus and cytoplasm of LNCaP cells treated with 10 nM DHT in time series of 0, 1, 2, 4, and 8 h. *KLK3* was used as a positive control. (**C**) The RIP assay detects the combination of *AC016745.3* and AR in LNCaP cells under 10 nM DHT stimulation in time series of 0, 1, and 4 h, respectively. *RP11-1023L17.1* served as a negative control. The result is the percentage of RNA in the Input. (**D**–**F**) The expression of AR target genes in LNCaP cells transfected with *AC016745.3* and treated with 10 nM DHT in time series of 2, 24, and 48 h. *KLK2*, *KLK3*, *KLK4*, *TMPRSS2*, and *NKX3-1*, are AR positive regulatory genes. *IRF1*, *PBX1* and *USO1* were negatively regulated by AR. Data are presented as the mean ± SD (n = 3). Significance was defined as *p* < 0.05 (*, *p* < 0.05; **, *p* < 0.01; ***, *p* < 0.001).

To investigate the possible molecular mechanism by which *AC016745.3* exerts its functions in PCa cells, the subcellular localization of this lncRNA was first examined. lncRNA *AC016745.3* expression was examined in the cytoplasmic and nuclear fractions of LNCaP cells that had been treated for different durations with 10 nM DHT (0, 1, 2, 4, and 8 h time points). qRT-PCR experiments show that lncRNA *AC016745.3* was present in both the nucleus and the cytoplasm following initial DHT stimulation, but by 2 h its expression was predominantly restricted to the nucleus (Figure 3A,B). Moreover, LncATLAS database analysis showed that *AC016745.3* was mainly distributed in the nucleus (Figure S1B). This suggests that *AC016745.3* may play a role in AR-dependent gene regulation.

**Figure 4:**
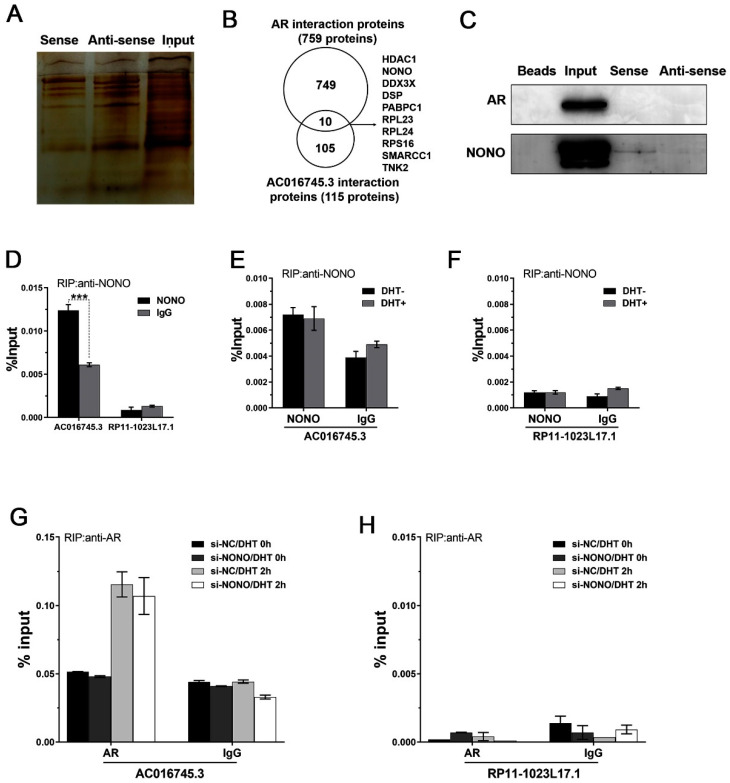
Screening for more *AC016745.3* and AR interacting proteins in AR transcription complex. (**A**) RNA pull-down assay analysis of *AC016745.3* bound proteins. The *AC016745.3* sense chain was transcribed in vitro, and its antisense chain was used as a control. After combination with the nuclear protein extracted by LNCaP, the binding protein was separated by Bis-Tris gel and stained with silver staining. Mass spectrometry results identified 115 proteins that specifically bind to the sense strand. (**B**) 115 proteins that bind to lncRNA *AC016745.3* from RNA pull-down and then intersect 759 AR-interacting proteins, thereby obtaining 10 candidate proteins that interact with both *AC016745.3* and AR. (**C**) Western blotting assay was used to verify the mass spectrometry results. Beads and Input were used as controls. (**D**) RNA immunoprecipitation (RIP) assay was used to verify the interaction between NONO and *AC016745.3*. (**E**,**F**) RIP assay was performed to detect the interaction between NONO and *AC016745.3* in LNCaP cells treated with 10 nM DHT for 2 h. (**G**,**H**) RIP assay was performed to detect the interaction between AR and *AC016745.3* in LNCaP cells transfected with *si-NONO* and treated with 10 nM DHT for 2 h. *RP11-1023L17.1* was used as a negative control. Data are presented as the mean ± SD (n = 3). The result is the percentage of RNA in the Input. Significance was defined as *p* < 0.05 (***, *p* < 0.001).

In order to investigate the possible role of *AC016745.3* in AR-dependent gene transcription, RNA immunoprecipitation (RIP) experiments were next performed to test whether *AC016745.3* is present in a complex with AR. These experiments revealed that, following DHT treatment, *AC016745.3* was significantly enriched in AR immunoprecipitates in LNCaP cells, indicating this lncRNA may have interacted with AR or AR complex in LNCaP cells (Figure 3C; Figure 4H). Subsequent qRT-PCR assays revealed that *AC016745.3* overexpression could partially suppress the expression of genes positively regulated by AR (*KLK2*, *KLK3*, *KLK4*, *TMPRSS2*, and *NKX3-1*), and partially derepress the expression of genes negatively regulated by AR (*IRF1*, *PBX1* and *USO1*), following cell treatment with DHT for 2 h (Figure 3D–F). Taken together, these findings indicate that *AC016745.3* inhibits the transcriptional activity of AR.

To explore whether *AC016745.3* affects the nuclear recruitment of AR, LNCaP cells were transfected with *AC016745.3* and then analyzed at different time points following treatment with 10 nM DHT. Western blotting analysis revealed that *AC016745.3* overexpression had no effect on the nuclear localization of AR (Figure S1E), indicating that *AC016745.3* does not block the nuclear recruitment of the receptor. In addition, ChIP-PCR assays showed that *AC016745.3* overexpression had no effect on the ability of AR to bind to the AREs of its target genes (Figure S1F,G). Taken together, these experiments demonstrate that *AC016745.3* can affect AR transcriptional activity without influencing the nuclear entry of the receptor or its ability to bind AREs within its target genes.

### 3.4. Identification of AC016745.3 and AR Interacting Proteins within the AR Transcription Complex

Even though the AR protein does not have a typical RNA binding domain, several lncRNAs have been reported to directly or indirectly interact with AR and regulate AR transcription activity [18,19,20]. It was hypothesized by the authors of this current study that *AC016745.3* may indirectly interact with AR through other AR binding partners. Therefore, the next step was to examine the possibility that *AC016745.3* affects AR transcriptional activity through its interactions with the other proteins of the AR transcriptional complex.

To investigate whether *AC016745.3* can affect AR transcriptional activity by impacting on proteins that interact with AR, the researchers first sought to identify the protein components of the AR transcriptional complex. From NCBI database (https://www.ncbi.nlm.nih.gov/gene/367 accessed on 19 October 2021), 759 proteins were identified that interacted with AR (Table S1). In parallel, RNA pull-down mass spectrometry assays were performed to identify proteins that form a complex with *AC016745.3*, and 115 protein binding partners were identified (Figure 4A; Table S2). There were 10 candidate proteins that were present in both protein data sets, and which, therefore, interacted with both *AC016745.3* and AR (Figure 4B). AR was not present in the *AC016745.3*-interacting protein data set, possibly because AR interacts with *AC016745.3* indirectly. In order to confirm the mass spectrometry analysis, we selected NONO for further validation by RNA pull-down and subsequent Western blotting analysis. These assays revealed that the *AC016745.3* sense strand can bind to NONO but not AR (Figure 4C). This finding supports the hypothesis that *AC016745.3* affects AR activity by interacting with AR binding proteins and further demonstrates lncRNA *AC016745.3* participated in the formation of an AR complex.

Of note, several previous studies had revealed NONO is an AR coregulator. RIP experiments were performed to further confirm the interaction between *AC016745.3* and NONO and AR. The results showed that *AC016745.3* was enriched in NONO immunoprecipitates in LNCaP cells (Figure 4D), and that DHT treatment did not influence this interaction (Figure 4E,F). These experiments also revealed that, in NONO knockdown cells, *AC016745.3* levels were slightly reduced in AR immunoprecipitates following DHT stimulation (Figure 4G,H). These results indicated NONO may have first interacted with *AC016745.3*, and then was recruited to the AR complex following DHT treatment, and then *AC016745.3* played a bridging role in recruiting other co-regulators.

### 3.5. NONO Is a Putative Proto-Oncogene That Promotes the Transcription of AR Target Genes

NONO possesses DNA and RNA binding domains, and directly interacts with the AF-1 region of AR. NONO also acts as an AR co-activator to promote AR-dependent transcriptional activity [21]. Therefore, it was next sought to determine whether *AC016745.3* affects the transcriptional activity of AR through its interactions with NONO.

**Figure 5:**
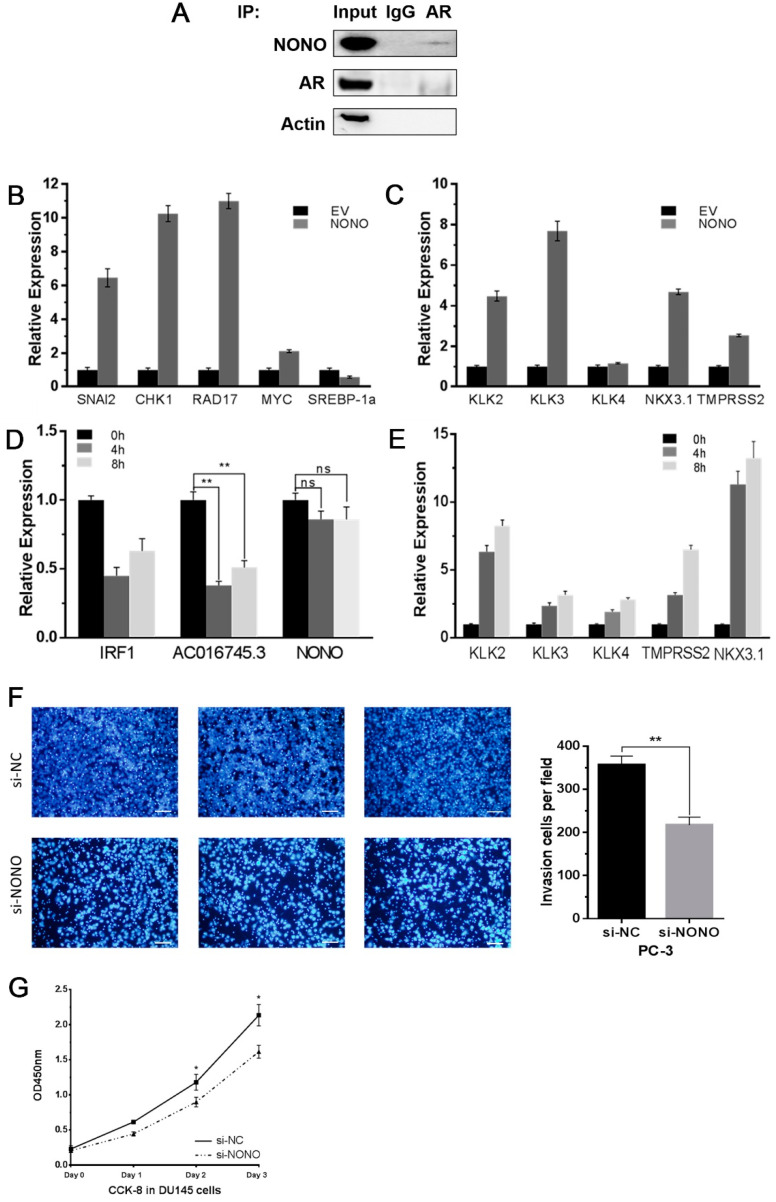
NONO promotes AR’s transcriptional regulation of its target genes as a proto-oncogene. (**A**) Co-IP experiments showed that AR can bind to NONO in LNCaP cells. Input and IgG were used as controls. (**B**,**C**) qRT-PCR was performed to detect the expression of target genes of NONO (**B**) and AR (**C**) after overexpressed NONO in LNCaP cells. (**D**,**E**) qRT-PCR was performed to detect the expression of *AC016745.3* and NONO in LNCaP cells treated with 10 nM DHT in time series of 0, 4, and 8 h. *IRF1*, *KLK2*, *KLK3*, *KLK4*, *NKX3.1*, and *TMPRSS2* are all target genes of AR and used as positive references. (**F**) Cell migration assay was performed in PC-3 cells by counting the amount of cells migrating in the transwell chamber. Scale Bar = 100 μm. (**G**) Cell proliferation analysis was performed with CCK-8 assay in DU145. Cells transfected with *si-NONO* were seeded into a 96-well plate at 5000 cells/well and examined at different time points (0, 1, 2, and 3 days). Data are presented as the mean ± SD (n = 3). Significance was defined as *p* < 0.05 (*, *p* < 0.05; **, *p* < 0.01).

Ishitani et al. reported that NONO can directly bind AR, and that DHT stimulation can promote this interaction [21]. Co-IP experiments also verified this interaction in LNCaP cells (Figure 5A). Overexpression of NONO in LNCaP cells promoted the expression of genes known to be positively regulated by NONO (*SNAI2*, *CHK1*, *RAD17*, and *MYC*), and inhibited the expression of *SREBP-1a*, a gene negatively regulated by NONO (Figure 5B). Moreover, NONO overexpression also promoted the expression of many genes positively regulated by AR (*KLK2*, *KLK3*, *NKX3.1*, and *TMPRSS2*), a finding that is in agreement with NONO’s role as an AR cofactor (Figure 5C). However, subsequent DHT stimulation experiments showed that *AC016745.3* expression was inhibited by DHT, and that this impaired the inhibitory effects of *AC016745.3* towards NONO and AR (Figure 5D,E). Conversely, NONO expression was not affected by DHT, suggesting that NONO expression may not be regulated by AR.

Next, cell phenotype experiments were performed to investigate the function of NONO in PCa cells. NONO knockdown significantly reduced PC-3 cell migration, when compared with the negative control (Figure 5F). Cell proliferation assays showed that inhibition of NONO expression also significantly inhibited the growth rate of DU145 cells (Figure 5G). Taken together, these findings suggest that NONO is a proto-oncogene that promotes the proliferation and metastasis of PCa cells.

### 3.6. AC016745.3 Partially Restores NONO Function in PCa Cells

**Figure 6:**
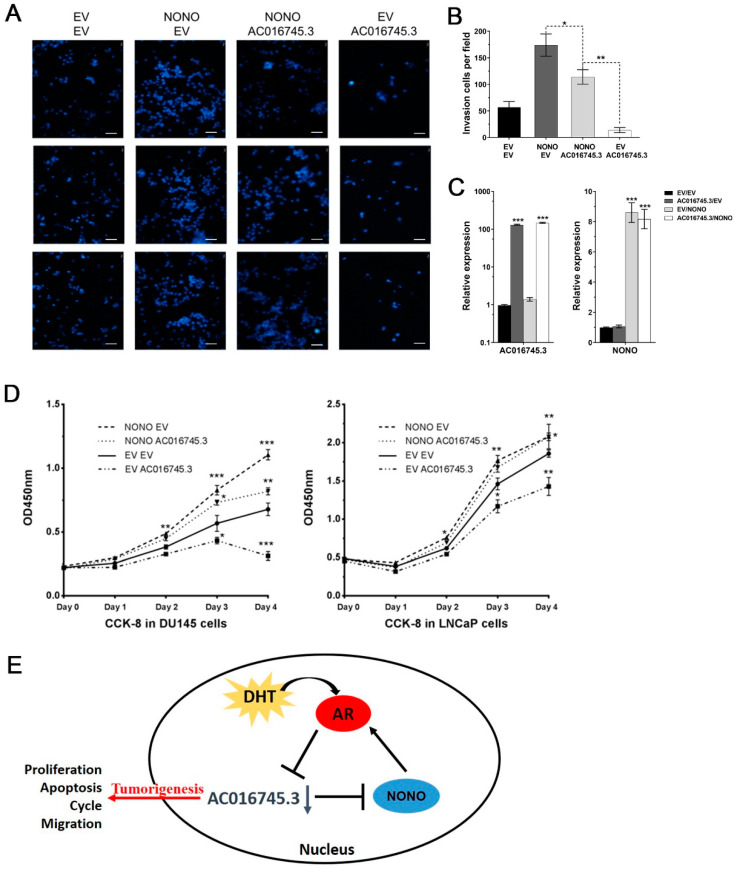
*AC016745.3* partially restores function of NONO in prostate cancer cells. (**A**,**B**) Cell migration assay was performed in PC-3 cells by counting the amount of cells migrating in the transwell chamber. Scale Bar = 100 μm. (**C**) qRT-PCR experiment was used to detect the transfection efficiency of NONO and *AC016745.3*. (**D**) Cell proliferation analysis was performed with CCK-8 assay in DU145 and LNCaP cells. Data are presented as the mean ± SD (n = 3). Significance was defined as *p* < 0.05 (*, *p* < 0.05; **, *p* < 0.01; ***, *p* < 0.001). (**E**) Schematic representation summarizing data from the present study.

To explore the relationship between NONO and *AC016745.3* at the cell phenotype level, overexpression experiments were performed and role of these factors was examined, alone or in combination, in the migration and proliferation of PCa cells. The transfection efficiency of the expression vectors used in the experiments is shown in Figure 6C. Cell phenotype experiments revealed that NONO transfection alone increased migration, and *AC016745.3* transfection alone reduced migration, and co-transfection of NONO and *AC016745.3* in PC-3 cells partially restored cells to control levels (Figure 6A,B). In DU145 and LNCaP cells, the co-transfection of NONO and *AC016745.3* partially restored to control levels both the accelerated cell proliferation caused by NONO transfection alone and the reduced cell proliferation caused by *AC016745.3* transfection alone (Figure 6D). Taken together, these findings suggest that *AC016745.3* expression can partially restore the function of NONO in PCa cells.

In summary, NONO is a co-activator of AR transcription. *AC016745.3* acts as a co-repressor of AR transcription through NONO binding. Under conditions of DHT stimulation, downregulation of *AC016745.3* suppresses the cell’s ability to inhibit AR transcriptional activity, thereby promoting PCa (Figure 6E).

## 4. Discussion

AR is a key transcription factor in the development and progression of PCa. Upon androgen stimulation, AR enters the nucleus and binds to AREs within target genes to regulate their transcription [2,3]. The current authors and other groups have shown that AR-regulated lncRNAs are associated with PCa carcinogenesis [22,23,24]. Building on previous research, this study has explored the potential role and mechanism of action of the AR target lncRNA *AC016745.3* in PCa. Cell phenotype experiments showed that *AC016745.3* overexpression inhibited the proliferation, promoted the apoptosis, and inhibited the migration of PCa cells. Moreover, this current study revealed lncRNA *AC016745.3* could suppress androgen receptor and its downstream signaling.

The formation of an AR transcriptional complex requires the functional and structural interaction of the AR with its coregulators. Recent studies have shown that some lncRNAs can interact with and influence the transcriptional activity of AR [12,13,14,15]. However, the molecular mechanism underlying this regulation remains unclear. This study’s preliminary experiments have shown that, under conditions of androgen stimulation, overexpression of *AC016745.3* inhibits the AR-dependent transcriptional regulation of target genes. In this study, it was found that *AC016745.3* levels were significantly enriched in AR immunoprecipitates in LNCaP cells following DHT treatment. An analysis of the AR interaction protein database and RNA pull-down mass spectrometry assay results showed that 10 proteins interacted with both *AC016745.3* and AR. These results demonstrated that *AC016745.3* indirectly interacts with AR, but participates in the formation of AR complex. Among these proteins, the focus was on NONO, which had been confirmed to be a known AR coregulator. NONO is a multifunctional nuclear protein that belongs to the *Drosophila* behavior human splicing (DBHS) protein family, and has been shown to be overexpressed in PCa [25,26]. Although NONO has been implicated in cancer development [27,28], its differential expression and functional involvement in PCa have not been fully explored. In this study, proteins that interact with *AC016745.3* were screened out, including NONO. NONO possesses conserved N-terminal RNA recognition motifs and a DNA binding domain [29,30]. NONO also directly interacts with the AF-1 region of AR, functioning as an AR co-activator to promote AR-dependent transcriptional activity. Therefore, it was hypothesized that *AC016745.3* interacts with NONO, thereby affecting the transcriptional activity of AR.

Ishitani et al. have reported that DHT can promote the interaction between NONO and AR [21]. In the current study, it was found that the interaction between *AC016745.3* and AR increases significantly following DHT stimulation, but that NONO binds *AC016745.3* independently of DHT. This finding demonstrates that, under conditions of DHT stimulation, *AC016745.3* binds to the AR transcription complex following its recruitment by NONO [21]. Consequently, it was surmised that *AC016745.3* antagonizes the activity of NONO. It was also found that *AC016745.3* expression was reduced under conditions of DHT stimulation, and that this impaired the antagonistic effects of *AC016745.3* towards NONO. Through cell phenotype experiments, it was found that *AC016745.3* can partially restore the cancer-promoting properties of NONO in PCa cells, suggesting that *AC016745.3* and NONO may antagonize one another in PCa.

The observation that *AC016745.3* only partially restores the function of NONO in PCa cells suggests that the cancer-promoting properties of NONO are only partly driven by AR activity, and that other AR/*AC016745.3*-independent mechanisms must also exist. Emerging evidence shows that NONO engages in almost every step of gene regulation, including, but not limited to, mRNA splicing, DNA unwinding, transcriptional regulation, nuclear retention of defective RNA, and DNA repair [31]. As such, there are diverse alternative mechanisms through which NONO may also exert its oncogenic functions.

These functional experiments found that *AC016745.3* and NONO still play a role in AR-negative prostate cancer cells. Our understanding is, that in addition to interacting with AR to affect the transcriptional activity of AR, NONO can also perform transcription activity in an AR-independent manner, and the interaction between *AC016745.3* and NONO does not depend on AR, therefore, they also play a role in AR-negative prostate cancer cells, but this is not the focus of this article. This work deserves further study in the future.

Several limitations of this study should also be noted. Firstly, the effect of *AC016745.3* on PCa apoptosis should be further validated using more assays. Secondly, the complicated regulatory relationship among AR/*AC016745.3*/NONO and other coregulators should be further confirmed, which will strengthen the findings of this study. Finally, while NONO has been confirmed to interact with *AC016745.3*, its detailed role in PCa remains largely unclear.

In conclusion, the following working model is proposed based on the results of this study (Figure 6E): NONO is a co-activator of AR under conditions of DHT stimulation, and *AC016745.3* negatively regulates the transcriptional activity of AR by binding to NONO. Following DHT stimulation, AR negatively regulates the expression of *AC016745.3*, thereby forming a negative feedback loop, which ultimately enhances AR activity and promotes tumorigenesis.

## Data Availability

Data is contained within the supplementary material.

## Supplementary Materials

The following are available online at https://www.mdpi.com/article/10.3390/life11111208/s1, Figure S1: Transfection efficiency and related verification, Table S1: Proteins interacted with AR acquired rom NCBI database, Table S2: RNA pulldown and mass spectrometry data in this study, Table S3: siRNA sequences used in knockdown experiments, Table S4: Primers used in qPCR, Table S5: Primers used in ChIP-PCR, Table S6: Primers used in Plasmid construction.
