# Supplementary material for: AC016745.3 Regulates the Transcription of AR Target Genes by Antagonizing NONO
Source: Life (Basel). 2021 Nov 9;11(11):1208. doi: 10.3390/life11111208 (PMC8625561; doi:10.3390/life11111208)
Supplement: Supplementary file 1 [file life-11-01208-s001.zip › life-1372663 SM.pdf]

Supplemantary Material

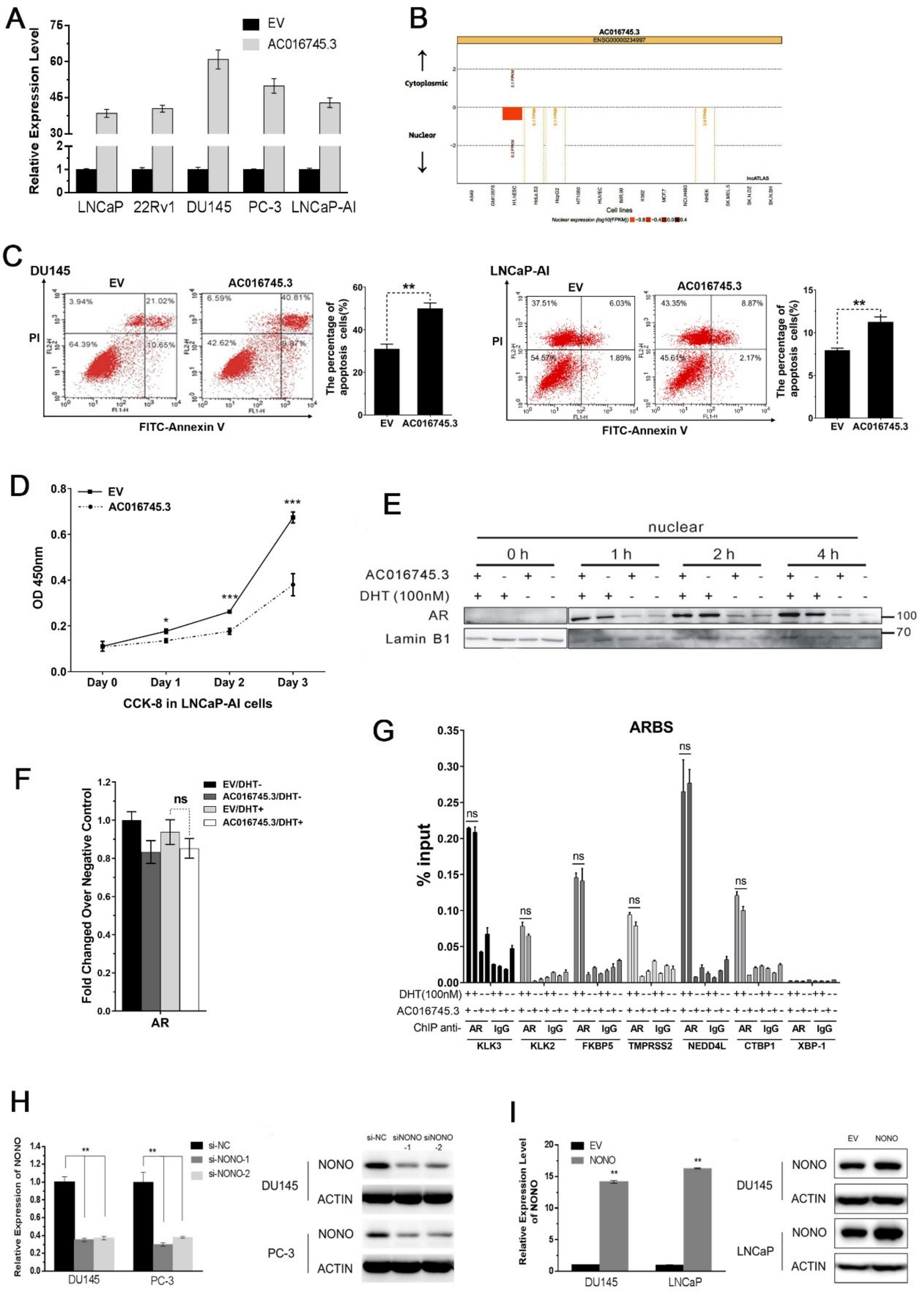


**Figure S1** (A) The transfection efficiency of high-expression plasmid pcDNA3.1- *AC016745.3* in prostate cancer cell lines. (B) The LncATLAS database was used to analyze the subcellular localization of *AC016745.3* in each cell line. (C) After highly expressing *AC016745.3* in prostate cancer cell lines DU145 and LNCaP-AI, stained with Annexin V/PI, and detected the changes of early and late apoptosis by FACScalibur flow cytometry. (D) After highly expressing *AC016745.3* in LNCaP-AI, the cell growth rate changes

were detected using CCK-8 Kit. (E) Western blot was used to detect AR entry into the nucleus after separation of nucleus and cytoplasm in LNCaP cells transfected with *AC016745.3* and treated with 10 nM DHT in time series of 0, 1, 2, and 4 h. Lamin B1 was used as a reference to the nucleus. (F) RT-PCR showed expression level of AR in LNCaP cells transfected with *AC016745.3* and treated with DHT. (G) ChIP-PCR assay detects the combination of AR and its ARE elements (*KLK3*, *KLK2*, *FKBP5*, *TMPRSS2*, *NEDD4L*, *CTBP1*, *XBP-1*) in LNCaP cells transfected with *AC016745.3* and treated with DHT. The result is the percentage of DNA in the input. (H-I) The efficiency of small interfering RNA of NONO and high-expression plasmid pcDNA3.1-NONO were confirmed by qRT-PCR and Western blot.

**Table S3.** siRNA sequences used in knockdown experiments.

| Name | Sequence（5′to 3′） |
|---|---|
| si-NC | UUCUCCGAACGUGUCACGUTT |
| si-AR-357 | GGAGCUCUCACAUGUGGAATT |
| si-AR-544 | GACAGUGUCACACAUUGAATT |
| si-NONO-1 | CCUAGCGGAGAUUGCCAAATT |
| si-NONO-2 | GCUCCUUUGAGUAUGAAUATT |

**Table S4.** Primers used in qPCR.

| Name | Sequence（5′to 3′） |
|---|---|
| β-actin-F | CCTCTCCCAAGTCCACACAG |
| β-actin-R | GGGCACGAAGGCTCATCATT |
| AC016745.3-F | GGTGACGAAAATTCTACATTGCT |
| AC016745.3-R | ACCTGGCTGGTGTCCTCTG |
| AR-F | GCCTTGCTCTCTAGCCTCAA |
| AR-R | GGTCGTCCACGTGTAAGTTG |
| RP11-1023L17.1-F | GATTTACTTTTGGGATACACTCATCAT |
| RP11-1023L17.1-R | ACAGGTGACTTCATCTTGGATTT |
| KLK2-F | ATCGAACCAGAGGAGTTCTTG |
| KLK2-R | CCCTTTCCCCTCCAGATGTT |
| KLK3-F | GTGCTTGTGGCCTCTCGT |
| KLK3-R | CAGCAAGATCACGCTTTTGT |
| TMPRSS2-F | ACTGGCTCCTGACTTAA |
| TMPRSS2-R | TTATGGCACTTGGCAATG |
| NKX3.1-F | CAGAGACCGAGCCAGAAAGG |
| NKX3.1-R | CTGAGTGTGGGAGAAGGCAG |
| KLK4-F | GAAGTGAGTGCAGAGCTCCTA |
| KLK4-R | GTTAGCGAGCAAGGGTCTGT |
| IRF1-F | GGCCCTGACTCCAGCTACAA |
| IRF1-R | TACCCCTTCCCATCCACGTT |
| PBX1-F | CCAACTCGGCTGGTGGATAC |
| PBX1-R | TGTACATCTGACTGGCTGCG |
| SNAI2-F | TTCGGACCCACACATTACCT |
| SNAI2-R | GAAAAAGGCTTCTCCCCCGTG |

| Name | Sequence（5′to 3′） |
|---|---|
| CHK1-F | TCTGCTCCTCTAGCTCTGCT |
| CHK1-R | TGGGAGACTCTGACACACCA |
| RAD17-F | TGGTGGGTCGGCTAGGAATA |
| RAD17-R | ACCATGAATTCACTTGCTCCT |
| MYC-F | TGGAAAACCAGCCTCCCG |
| MYC-R | TTCTCCTCCTCGTCGCAGTA |
| SREBP-1a-F | GCTCCCTAGGAAGGGCCGTA |
| SREBP-1a-R | CGCCGACTTCACCTTCGAT |
| USO1-F | AAGATCGACAGACGGGGAAG |
| USO1-R | AGGGCACATAAGCCTTGGAC |
| NONO-F | ACAGATGCAGTGAAGGCTC |
| NONO-R | CTCGTTCCTTGTGAAATTGCTG |

**Table S5.** Primers used in ChIP-PCR.

| Name | Sequence（5′to 3′） |
|---|---|
| ARE1-F | TACATCATAGACCTCCCAAACAG |
| ARE1-R | GGGAGAGGGACCATTATCTGGC |
| ARE2-F | CCCTAGGATAGATTTCTTGAAGA |
| ARE2-R | TTTTATTGTAGTTTTGGTTGCATAA |
| PSA enhancer-F | TGGGACAACTTGCAAACCTG |
| PSA enhancer-R | CCAGAGTAGGTCTGTTTTCAATCCA |
| miR-125b-F | TACAATTCCTGTGAAATGAGAGCAC |
| miR-125b-R | TTCTATGTGATAATGAAAAAAACCCTA |
| XBP1-promoter-F | TCTGGAAAGCTCTCGGTTTG |
| XBP1-promoter-R | AATCCCTGGCCAAAGGTACT |
| KLK2-F | TGTGTGCAGGAAGAGCGGGTGAA |
| KLK2-R | ACCTGACCCTCCATTCTAACTGGT |
| KLK3-F | TGGGACAACTTGCAAACCTG |
| KLK3-R | CCAGAGTAGGTCTGTTTTCAATCCA |
| FKBP5-F | ATTATCCGGAGAACCAAACG |
| FKBP5-R | ACATCCTTCCACCACAG |
| TMPRSS2-F | CAGGAGTGTACGGGAATGTGATGGT |
| TMPRSS2-R | GATTAGCCGTCTGCCCTCATTTGT |
| NEDD4L-F | CAACTTGGACTCGGCCAATC |
| NEDD4L-R | GTTACTGTTGGCGAGCTGAG |
| CTBP1-F | ACACCCAAGGTCCTCAG |
| CTBP1-R | TCCTTTGTTCACAGAGTCAG |

**Table S6.** Primers used in Plasmid construction.

| Name | Sequence（5′to 3′） |
|---|---|
| AC016745.3-clone-F | AGCTGGGAAAACGGC |
| AC016745.3-clone-R | ATAACAGAACCTATACTATAAAATGG |
| AC016745.3-T7-F | TAATACGACTCACTATAGGGAGCTGGGAAAACGGC |
| AC016745.3-T7-R | TAATACGACTCACTATAGGGATAACAGAACCTATACTATAAAATGG |
| NONO-clone-F | ATGCAGAGTAATAAAACTTTTAACTTGGA |
| NONO-clone-R | TTAGTATCGGCGACGTTTGTTTG |
